# Triplet chemotherapy with vinorelbine, gemcitabine, and cisplatin for advanced non-small cell lung cancer: a phase II study

**DOI:** 10.1038/sj.bjc.6600658

**Published:** 2002-11-26

**Authors:** S Niho, K Kubota, K Goto, H Ohmatsu, T Matsumoto, R Kakinuma, Yutaka Nishiwaki

**Affiliations:** Division of Thoracic Oncology, National Cancer Center Hospital East, Chiba, Japan

**Keywords:** triplet, vinorelbine, gemcitabine, cisplatin, non-small cell lung cancer (NSCLC)

## Abstract

We conducted a phase II trial of triplet chemotherapy consisting of vinorelbine, gemcitabine, and cisplatin in patients with advanced non-small cell lung cancer to assess its efficacy and toxicity. Thirty-three patients with chemotherapy-naïve stage IIIB disease (*n*=8), stage IV disease (*n*=23), or recurrence after surgical resection (*n*=2) were given intravenous infusions of vinorelbine 25 mg m^−2^, gemcitabine 1000 mg m^−2^, and cisplatin 40 mg m^−2^ on days 1 and 8 at 3-week intervals. There were 16 partial responses, and the objective response rate was 48% (95% confidence interval: 31–66%). The median survival time was 13.5 months (95% confidence interval: 10.6–16.4 months), and the one-year survival rate was 61%. Grade 4 haematologic toxicity consisted of neutropenia in 72% of patients, and febrile neutropenia occurred in 42% of the patients. There was one toxic death, and it was attributed to neutropenic fever and haemoptysis. Autopsy revealed diffuse pulmonary haemorrhage secondary to bacterial abscesses and vasculitis in both lungs. The common nonhaematologic toxicities included grade 2–3 nausea (39%) and vomiting (18%). Triplet chemotherapy containing vinorelbine, gemcitabine, and cisplatin is effective in the treatment of chemo-näive patients with advanced non-small cell lung cancer, but produces unacceptable frequent febrile neutropenia.

*British Journal of Cancer* (2002) **87**, 1360–1364. doi:10.1038/sj.bjc.6600658 www.bjcancer.com

© 2002 Cancer Research UK

## 

Lung cancer is a leading cause of cancer-related deaths in industrialised countries, including Japan, where 54 000 people died of lung cancer in 2000. Non-small cell lung cancer (NSCLC) accounts for approximately 80% of lung cancers, and approximately 70% of patients present with locally advanced or metastatic disease. Meta-analysis demonstrated that cisplatin-based chemotherapy yielded a modest survival benefit in advanced NSCLC (Grilli *et al*, 1993; Souquet *et al*, 1993; Non-Small Cell Lung Cancer Collaborative Group, 1995; Marino *et al*, 1994).

In the 1990's, several new agents, including vinorelbine, gemcitabine, paclitaxel, docetaxel, and irinotecan, became available for the treatment of NSCLC. Vinorelbine or gemcitabine combined with cisplatin was found to result in longer survival than cisplatin alone or vindesine plus cisplatin (Le Chevalier *et al*, 1994; Wozniak *et al*, 1998; Sandler *et al*, 2000). Paclitaxel plus cisplatin was also superior to etoposide plus cisplatin (Bonomi *et al*, 2000). The Southwest Oncology Group (SWOG) conducted a randomised phase III trial in advanced NSCLC to compare vinorelbine plus cisplatin with paclitaxel plus carboplatin (Kelly *et al*, 2001), but there was no significant difference between the two arms with respect to response rate, survival, or quality of life. The Eastern Cooperative Oncology Group (ECOG) conducted a randomised phase III trial in advanced NSCLC comparing three platinum-based combination regimens containing gemcitabine, paclitaxel, and docetaxel to a reference regimen of paclitaxel plus cisplatin (Schiller *et al*, 2002), but none of the regimens, i.e. gemcitabine plus cisplatin, paclitaxel plus carboplatin, docetaxel plus cisplatin yielded longer survival than paclitaxel plus cisplatin. Irinotecan plus cisplatin yielded longer survival than vindesine plus cisplatin in stage IV NSCLC (Fukuoka *et al*, 2000). As a result, each of these new agents plus cisplatin and paclitaxel plus carboplatin have become the standard regimens for advanced NSCLC, but survival with these doublet chemotherapies is not satisfactory.

Triplet chemotherapy or sequential chemotherapy with more than two agents has been investigated to surpass standard doublet chemotherapy. Triplet chemotherapy containing old agents has never been superior to doublet chemotherapy in advanced NSCLC. The activity of the new agents, however, has encouraged the development and evaluation of new triplet combinations. Recently, Comella *et al* (2000, 2001) reported promising activity and safety of triplet combination including cisplatin, vinorelbine, plus gemcitabine, and cisplatin, gemcitabine, plus paclitaxel. These triplet combinations yielded better survival than cisplatin plus vinorelbine and cisplatin plus gemcitabine. Cisplatin, vinorelbine, plus gemcitabine was followed by a median survival time of 51 weeks, a 45% 1-year survival rate, and 45% grade 3 or 4 neutropenia with no toxic deaths. We considered it necessary to evaluate this regimen with respect to its efficacy and toxicity profile. The primary end point of this phase II trial was response rate, and the secondary end points were toxicity and survival.

## PATIENTS AND METHODS

### Patient population

Patients were required to have histologically or cytologically confirmed stage IIIB (no indications for curative thoracic radiation therapy for malignant pleural or pericardial effusion and/or a too wide radiation field) or stage IV NSCLC. Recurrences after surgical resection were permitted. Other criteria included: (1) age 20 years or more but less than 75 years; (2) ECOG performance status (PS) 0 or 1; (3) measurable disease; (4) adequate organ function (i.e., total bilirubin ⩽1.1, AST and ALT ⩽60 IU l^−1^, serum creatinine ⩽1.2 mg dl^−1^, creatinine clearance ⩾60 ml min^−1^, leukocyte count 4000–12 000 mm^−3^, neutrophil count ⩾2000 mm^−3^, haemoglobin ⩾9.0 g dl^−1^, and platelets ⩾100 000 mm^−3^); (5) no prior chemotherapy or radiotherapy; (6) no severe heart disease or uncontrolled angina; (7) no cardiac infarction within the previous 6 months; (8) no uncontrolled diabetes mellitus; (9) no active infection; (10) no active concomitant malignancy; (11) no pregnancy or breast-feeding. No palliative bone or brain radiotherapy was allowed. All patients were required to provide written informed consent, and the institutional review board at the National Cancer Center approved the protocol.

### Treatment plan

Treatment was started within a week after enrolment in the study. Patients received vinorelbine (25 mg m^−2^) diluted in 50 ml normal saline as a 5- to 10-min intravenous infusion with 16 mg of dexamethasone and 3 mg of granisetron immediately prior to vinorelbine infusion. Immediately after completion of the vinorelbine infusion, flushing was performed with 100 ml normal saline over 20 min to prevent phlebitis. Gemcitabine (1000 mg m^−2^) diluted in 100 ml normal saline was then intravenously infused over 30 min. Finally, cisplatin (40 mg m^−2^) was administered with 1500 ml of normal saline as a 30-min intravenous infusion over 3½ h. Vinorelbine, gemcitabine, and cisplatin were administered on days 1 and 8. Treatment was repeated every 3 weeks. None of the drugs was administered if WBC <2500 mm^−3^ and/or platelets <50 000 mm^−3^. Only vinorelbine and gemcitabine were administered if serum creatinine >1.5 mg dl^−1^. In the event of grade 4 leukopenia or thrombocytopenia, non-haematologic grade 3 or more toxicities, and/or omission of the treatment on day 8, the doses of all three drugs were reduced by 20% in the next course of chemotherapy. If serum creatinine increased to 2.0 mg dl^−1^ or more, only cisplatin was reduced, by 20%, in the next course of chemotherapy. The next course of chemotherapy was started if the following criteria were met: WBC ⩾3000 mm^−3^, platelets ⩾75 000 mm^−3^, AST and ALT ⩽2 times the upper limit of normal, ECOG PS 0-2, and afebrile. Vinorelbine and gemcitabine alone were administered in the next course if only the serum creatinine criterion was not satisfied after delaying the next course for up to 6 weeks. Therapy was continued for at least three courses unless the patient experienced unacceptable toxicity or had progressive disease. The maximum number of courses was six.

### Study evaluations

Pretreatment evaluations consisted of a complete medical history, determination of performance status, physical examination, haematologic and biochemical profiles, electrocardiogram, chest X-ray, bone scan, and computed tomography (CT) scan of the chest, ultrasound or CT scan of the abdomen, and magnetic resonance imaging (MRI) or CT scan of the whole brain. Evaluations performed weekly were biochemistry, complete blood cell, platelet, and leukocyte differential counts, physical examination, determination of performance status, and toxicity assessment. Imaging studies were performed to assess objective response after every two treatment courses.

### Response and toxicity criteria

Response Evaluation Criteria in Solid Tumors (RECIST) guidelines were used (Therasse *et al*, 2000). The target lesions were defined as ⩾2 cm in longest diameter on computed tomographic scans. A complete response (CR) was defined as the complete disappearance of all clinically detectable tumours for at least 4 weeks. A partial response (PR) was defined as an at least 30% decrease in the sum of the longest diameters of the target lesions for more than 4 weeks with no new area of malignant disease. Progressive disease (PD) indicated at least a 20% increase in the sum of the longest diameter of target lesions or a new malignant lesion. Stable disease (SD) was defined as insufficient shrinkage to qualify for PR and insufficient increase to qualify for PD. Toxicity was graded according to the National Cancer Institute Common Toxicity Criteria (NCI-CTC) version 2.0.

### Statistical analysis

In accordance with the minimax two-stage phase II study design by Simon (1989), the treatment program was designed to refuse response rates of 20% (P_0_) and to provide a significance level of 0.05 with a statistical power of 80% in assessing the activity of the regimen as a 40% response rate (P_1_). The upper limit for first-stage drug rejection was four responses in the 18 evaluable patients; the upper limit of second-stage rejection was 10 responses in the 33 evaluable patients. Overall survival was defined as the interval between enrollment in this study and death or the final follow-up visit. Median overall survival was estimated by the Kaplan–Meier analysis method (Kaplan and Meier, 1958).

## RESULTS

### Patient population and treatment

A total of 33 patients were enrolled in this study between March 31, 2000 and September 25, 2000. Patient characteristics are listed in Table 1

**Table 1:**
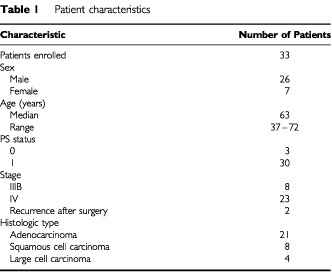
Patient characteristics

. The majority of patients were male (79%); median age was 63 years. The most common histologic subtype was adenocarcinoma (64%). Most patients (76%) had stage IV disease or recurrence after surgical resection.

A total of 115 courses were administered. The median number of treatment courses was three (range: 1–6). Thirteen patients experienced dose reduction because of omission of the treatment on day 8 (*n*=7) and neutropenic fever grade 3 (*n*=6).

### Efficacy

There were 16 partial responses in 33 eligible patients and the objective response rate was 48% (95% confidence interval (CI): 31–66%) (Table 2

**Table 2:**
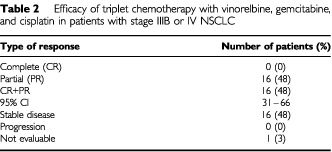
Efficacy of triplet chemotherapy with vinorelbine, gemcitabine, and cisplatin in patients with stage IIIB or IV NSCLC

). As described below, one patient was not evaluable for response because of early death.

**Figure 1:**
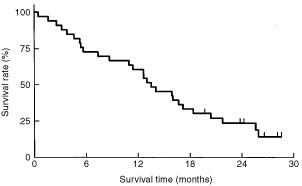
Overall survival of all patients (*n*=33) was calculated according to the Kaplan–Meier method. The median survival time was 13.5 months (95% CI: 10.6–16.4 months), and the one-year survival rate was 61%

The median follow-up time was 25 months, and six patients were still alive at the most recent follow up. The median time to response was 1.4 months (range 0.6–2.6 months). The median duration of response was 3.5 months (1.2–15.6 months). The median time to disease progression was 5.0 months (95% CI: 3.7–6.3 months). The median survival time was 13.5 months (95% CI: 10.6–16.4 months), and the one-year survival rate was 61% (Figure 1

).

### Safety and toxicity

Toxicity was evaluated in all enrolled patients and in all cycles. The most common toxicity was haematologic (Table 3

**Table 3:**
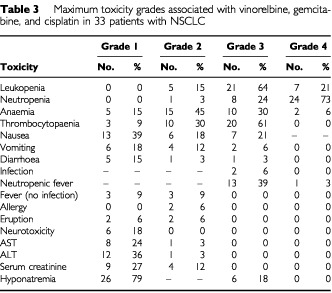
Maximum toxicity grades associated with vinorelbine, gemcitabine, and cisplatin in 33 patients with NSCLC

). Grade 4 neutropenia occurred in 72% of the patients and 35% of the courses. Febrile neutropenia occurred in 42% of patients. There was one toxic death, attributed to neutropenic fever and haemoptysis, in a 68-year-old male. The pre-treatment blood studies showed a leukocyte count of 8600 mm^−3^, platelet count of 225 000 mm^−3^, and serum creatinine level of 1.0 mg ml^−1^. The blood studies on day 8 showed a leukocyte count of 2900 mm^−3^, platelet count of 84 000 mm^−3^, and serum creatinine level of 1.4 mg dl^−1^. These levels met the criteria, and the agents were administered on day 8. On day 11, the leukocyte count was 3200 mm^−3^, and the platelet count 49 000 mm^−3^. Intravenous fluids had been administered on day 13 because of nausea and poor food intake. On day 14, the leukocyte count fell to 800 mm^−3^ with a neutrophil count of 60 mm^−3^, the platelet count was 29 000 mm^−3^, and serum creatinine level was 2.8 mg ml^−1^. Fever, haemoptysis, and dyspnoea developed, and arterial blood gas analysis showed a PaO_2_ of 45.7 torr. Transbronchial intubation was performed to allow artificial ventilation. The respiratory failure worsened, and the patient died on day 14. Autopsy revealed diffuse pulmonary haemorrhage secondary to bacterial abscesses and vasculitis in both lungs.

Grade 3 nausea was observed in seven patients (21%) and required intravenous infusions. Grade 2 creatinine elevation was observed in four patients (12%). Vinorelbine and gemcitabine alone were administered to one patient during courses 5 and 6 because of serum creatinine elevation to above 1.5 mg dl^−1^. Performance status deteriorated in 16 patients, and chemotherapy was terminated in five of them because of poor performance status.

### Second-line chemotherapy

A total of 13 patients received second-line chemotherapy. Eleven patients received docetaxel-based chemotherapy. One patient received ZD-1839 (Iressa®), a tyrosine-kinase inhibitor. The remaining patient received the same regimen of chemotherapy (vinorelbine, gemcitabine, and cisplatin).

## DISCUSSION

We reported a phase II study designed to evaluate the efficacy and safety of triplet chemotherapy containing vinorelbine, gemcitabine, and cisplatin in patients with advanced NSCLC. There are several reports on this triplet chemotherapy, mainly in Europe (Table 4

**Table 4:**
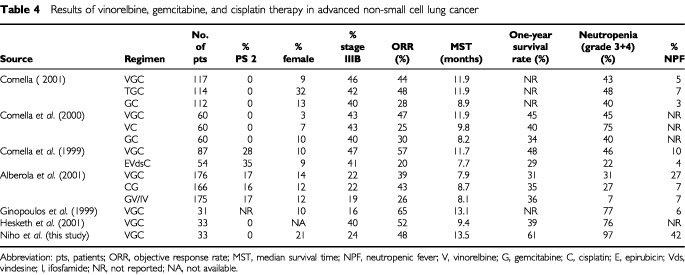
Results of vinorelbine, gemcitabine, and cisplatin therapy in advanced non-small cell lung cancer

). Italian and Spanish trials showed that grade 3 or 4 neutropenia occurred in 30% to 45% of patients, and that neutropenic fever occurred in 5–27% of patients (Comella *et al*, 1999, 2000; Alberola *et al*, 2001; Comella, 2001). In our own trial, however, grade 3 or 4 neutropenia and neutropenic fever occurred in 97% and 42%, respectively, of patients. This frequent febrile neutropenia is not acceptable. In the Italian trials, full doses of chemotherapy were given if the neutrophil and platelet counts on the day of treatment were ⩾2000 mm^−3^ and ⩾100 000 mm^−3^, and chemotherapy was given at 75% of the planned dose if grade 1 myelotoxicity (except anaemia) on the World Health Organization (WHO) grade scale was present on day 8. Grade 1 myelotoxicity on the WHO grade scale means leukocyte, neutrophil, and platelet counts of 3000–3999 mm^−3^, 1500–1999 mm^−3^, and 75 000–99 999 mm^−3^, respectively (Miller *et al*, 1981). By contrast, our criteria for administration of the agents on day 8 were a leukocyte count of 2500 mm^−3^ and a platelet count of 50 000 mm^−3^ or more. Grade 3 or 4 neutropenic fever occurred in 14 of the 33 patients (42%) and in 17 of the 115 courses (15%). Leukocyte counts less than 2500 mm^−3^ on day 8 of the course occurred in 16 courses, counts of 2500–3000 mm^−3^ in 20 courses, and of 3000 mm^−3^ or more in 79 courses. Grade 3 or 4 neutropenic fever was observed in 0 (0%), 6 (30%), and 11 (14%) of these courses, respectively. The criteria should be changed to leukocyte counts of 4000 mm^−3^ or more for full doses of the agents and 3000–3999 mm^−3^ for 75% doses. If the leukocyte and platelet counts on day 8 do not meet the criteria, chemotherapy should be postponed to day 15. These differences in criteria for administration of the agents on day 8 seemed to be responsible for the severe neutropenia and neutropenic fever in our trial.

The 48% response rate in our study was equivalent to the rate in the previous study. The 13.5-month median survival time and 61% 1-year survival rate in our study seem higher than in previous studies (Comella *et al*, 1999, 2000; Ginopoulos *et al*, 1999; Alberola *et al*, 2001; Comella, 2001; Hesketh *et al*, 2001), and the differences are probably attributable to patient selection. Female patients accounted for 21% in our trial, as opposed to only 3–14% in the European trials (Alberola *et al*, 2001; Comella *et al*, 1999, 2000, Comella, 2001; Ginopoulos *et al*, 1999). The female patients survived statistically longer than the males in our trial, with 1-year survival rates of 86% and 54%, respectively (*P*=0.035, log-rank test). No PS 2 patients were included in our trial. On the other hand, the Spanish trial included 14% of PS 2 patients. The Italian trial demonstrated that triplet chemotherapy containing cisplatin, gemcitabine, and vinorelbine or paclitaxel was superior to standard doublet chemotherapy containing cisplatin and vinorelbine, or gemcitabine (Comella *et al*, 2000; Comella, 2001). The Spanish trial, however, did not confirm the superiority of either triplet chemotherapy (Alberola *et al*, 2001). More randomised trials comparing triplets containing new agents with their corresponding doublets are warranted.

One patient developed life-threatening haemoptysis. Autopsy demonstrated diffuse pulmonary haemorrhage due to bacterial abscesses and vasculitis in both lungs. As far as we have been able to determine, this is the first report of haemoptysis after administration of vinorelbine, gemcitabine, and cisplatin.

Cisplatin was administered on days 1 and 8 in our trial and the Italian trial, whereas 75–100 mg m^−2^ was administered on day 1 in the Spanish and Greek trial (Ginopoulos *et al*, 1999; Alberola *et al*, 2001). Divided doses of cisplatin reduce the volume of fluid infused for hydration and make administration to outpatients more convenient.

In conclusion, triplet chemotherapy containing vinorelbine, gemcitabine, and cisplatin is effective in the treatment of chemotherapy-näive patients with advanced NSCLC, but produces unacceptable frequent febrile neutropenia. This triplet regimen should not be taken forward.
